# Reformulating Cookies with Colored Whole Wheat Flours and Xylitol: Implications for Technological Quality, Phenolic Content, and Glycemic Response

**DOI:** 10.3390/foods15071244

**Published:** 2026-04-05

**Authors:** Fazilet Mıdık, Kubra Ozkan, Lale Karataylioglu, Cagla Ozer, Vladimir P. Shamanin, Inna V. Pototskaya, Alexey I. Morgounov, Osman Sagdic, Hamit Koksel

**Affiliations:** 1 Department of Gastronomy and Culinary Arts, Faculty of Fine Arts, Design and Architecture, Istinye University, 34396 Istanbul, Türkiye; fazilet.midik@istinye.edu.tr (F.M.); lale.karataylioglu@istinye.edu.tr (L.K.); cozer@istinye.edu.tr (C.O.); 2 Department of Food Engineering, Faculty of Chemical and Metallurgical Engineering, Yildiz Technical University, 34349 Istanbul, Türkiye; kubraozkan1907@gmail.com (K.O.); osagdic@yildiz.edu.tr (O.S.); 3 Department of Agronomy, Breeding and Seed Production of the Agrotechnological Faculty, Omsk State Agrarian University, 1 Institutskaya pl., Omsk 644008, Russia; vp.shamanin@omgau.org (V.P.S.); iv.pototskaya@omgau.org (I.V.P.); 4 Department of Botany and Agroecology, Al Farabi Kazakh National University, Almaty 050051, Kazakhstan

**Keywords:** colored wheat, cookie, texture, glycemic index, phenolic compounds, antioxidant capacity

## Abstract

This study investigated the effects of incorporating colored wheat flours (red, blue, purple, and black) and replacing sucrose with xylitol on the technological, functional, and nutritional properties of cookies. Cookies were produced using 50:50 blends of colored whole wheat flours and refined cookie flour, and their physical, color, textural, phenolic, antioxidant, and in vitro glycemic index (GI) properties were evaluated. It has been determined that the addition of colored wheat flours significantly alters the textural properties. The incorporation of colored wheat flours significantly decreased width and increased thickness compared with the control cookies. The spread ratio of sucrose-containing cookies was higher (5.07 to 5.82) compared to xylitol-containing ones (4.91 to 5.41). Substitution of sucrose with xylitol generally reduced dough hardness. The colored wheat flour cookies had lower lightness values (52.31 to 63.18) compared to control samples (68.38 and 69.07 for sucrose and xylitol), while xylitol-based formulations produced slightly lighter cookies due to their lower browning potential. The cookies containing colored whole wheat flours exhibited higher hardness and brittleness than control cookies, likely due to their higher dietary fiber content, whereas xylitol resulted in softer cookies than sucrose. Cookies prepared with colored wheats showed significantly higher total phenolic content (367.41 and 424.87 mg GAE/100 g) and antioxidant capacity than the control samples (312.42 and 306.28 mg GAE/100 g for sucrose and xylitol), with purple wheat cookies exhibiting the highest values (424.69 and 424.87 mg GAE/100 g for sucrose and xylitol). Furthermore, colored wheat cookies demonstrated lower estimated GI values compared with control cookies (73.74 and 67.12), particularly those produced with blue wheat (66.68 and 60.94). Overall, the results indicate that colored wheat flours combined with alternative sweeteners such as xylitol can be used to develop cookies with improved antioxidant properties and moderated glycemic response while maintaining acceptable technological quality.

## 1. Introduction

Colored wheat varieties, including purple, blue, and black wheat, have attracted recognizable attention due to their enriched profiles of bioactive compounds like anthocyanins, carotenoids, and phenolic acids, which are primarily concentrated in the outer layers of the grain [1,2,3,4,5]. Besides the functional contributions of pigments found in their bran layer, colored wheats are also considered as potential replacements for synthetic dyes by the food industry [4].

These phytochemicals exhibit potent antioxidant and anti-inflammatory properties, contributing to the prevention of chronic diseases such as obesity, type 2 diabetes, cardiovascular disease, and certain cancers [3,5,6,7]. Accordingly, colored wheat is gaining popularity in the development of functional bakery and cereal-based products that offer both nutritional and health-promoting benefits [1,2,4,8].

Cookies are popular snack foods largely because of their convenience and palatability [2,9]. Nevertheless, conventional cookies are typically high in fat and added sugars while being low in dietary fiber, minerals, and other functional constituents, and they generally exhibit a high glycemic index (GI) due to their formulation with refined wheat flour. As a result, excessive consumption may adversely affect human health. Growing consumer awareness of diet–health relationships has driven increased interest in cookies with enhanced nutritional and functional attributes. In this context, cookies represent a suitable vehicle for the development of value-added foods as their nutritional profile can be effectively improved through flour replacement or enrichment with health-promoting ingredients. There is a growing trend toward replacing table sugar with sugar alcohol in cookie formulations, with the aim of lowering GI and making these products more suitable for health-conscious consumers and those managing blood glucose levels [5,10,11].

Despite the promising health attributes of colored wheats, there are still gaps in the literature regarding their optimal use in cookie formulations, particularly in the impact of sugar alcohol substitution and the positive contribution of phenolic content on product quality [1,2,4,9]. Previous research on cookies formulated with colored wheats (such as purple, blue, or black wheats) is available in the context of health benefits associated with anthocyanins and other phenolic compounds present in these grains, which can enhance antioxidant activity and nutritional value in bakery products [12,13,14,15]. On the other hand, xylitol is a commonly used alternative offering lower glycemic impact and improved, stable storage capacity [16,17,18]. However, a comprehensive review of the literature reveals that while cookies made with colored wheat and those containing xylitol have been studied separately, published studies combining both ingredients in cookie formulations are limited. Some of the studies also focused on consumer acceptance and investigated the sensory attributes of cookies produced using colored wheats [1,9,16]. This research aimed to meet the growing demand from consumers for healthier baked goods. To this end, the potential for producing cookies with improved functional properties and a lower glycemic index using a combination of colored whole wheat flours was investigated.

## 2. Materials and Methods

### 2.1. Materials

Wheat grains of four colors (red, purple, blue, black) were obtained from the plots grown at Omsk State Agrarian University (Omsk, Russia) in 2024. The following genotypes were used in the study: red-variety Element 22 (Granit/Saratovskaya 29/3/Erythrospermum 59//Tselinnaya 20/Tertsiya), purple-variety Omskiy ametist (Element 22 *2/i:S29PF), blue-Line 101-23 (Stolypin kaya 2//S29_ (4Th/4D)/Element 22), and black—Line 241-21 (Element 22 *2/S29_ (4Th/4D)//Element 22 *2/i:S29PF). Cookie flour was supplied from a cookie processing company (Ulker Co., Ankara, Türkiye). All-purpose shortening, high-fructose corn syrup 42%, ammonium bicarbonate, and xylitol (99.5% purity from corncob) were supplied from Aymar Yağ ve Gıda Inc. (Istanbul, Türkiye), PNS Pendik Nişasta Inc. (Istanbul, Türkiye), and Pastaland Inc. (Istanbul, Türkiye), Kimbiotek Inc. Co. (Istanbul, Türkiye), respectively. Other ingredients (sucrose, nonfat dry milk, salt, and sodium bicarbonate) were purchased from supermarkets in Istanbul.

### 2.2. Methods

#### 2.2.1. Milling

Whole wheat flours were produced in accordance with AACC Method 26-21 [19] using a Bühler MLU 202 pneumatic laboratory mill (Bühler, Uzwil, Switzerland), as previously described by Koksel et al. [12]. During the milling process, both coarse and fine bran fractions were collected and subsequently re-ground using a Perten 3100 laboratory mill (Perkin Elmer, Shelton, CT, USA) equipped with a 500 μm sieve to ensure uniform particle size. The reconstituted whole wheat flour was obtained by thoroughly blending the corresponding endosperm and bran fractions from each colored wheat genotype [12].

#### 2.2.2. Cookie Production

A 50:50 blend (*w*/*w*) of cookie flour and whole wheat flour of the colored genotypes was used to formulate two types of cookie samples: with sucrose or xylitol. All samples were prepared in parallel with duplicates per group, and 100% cookie flour with sucrose and 100% cookie flour with xylitol were used as control samples.

The model cookies were prepared according to the AACC (American Association of Cereal Chemists) International Method No. 10-54.01 (Baking Quality of Cookie Flour-Micro Wire-Cut Formulation), with some modifications, to study the effects of two different types of sugar and four colored wheat flours [19].

The recipe contained a blend of 20.0 g of whole wheat flour (from one of the colored wheats) and 20.0 g of cookie flour, 16.8 g of sucrose or xylitol, 16.0 g of all-purpose shortening, 0.5 g of sodium chloride, 0.4 g of sodium bicarbonate, 0.6 mL high-fructose syrup, 0.4 g of non-fat dry milk, and 8.8 mL of water.

The baking process was carried out in an industrial oven (Electrolux, Skyline Premiums 6 GN 1/1, Stockholm, Sweden) at 203 ± 2 °C for 8 min. The baked cookies were allowed to cool at room temperature. Once cooled, they were wrapped in aluminum foil and stored at room temperature until further analysis was conducted.

#### 2.2.3. Quality Parameters of Cookies

Following the cooling period, the width (W) and thickness (T) of the cookie samples were measured using a digital caliper Model SL01 (Deqing Shengtaixin Electronic Technology Co., Ltd., Hangzhou, China). The spread ratio was calculated by dividing the width by the thickness (W/T), providing an indication of the cookie’s dimensional spread during baking. All experiments were calculated according to AACC Method No. 10-50.05 and No 10-54.01 [19].

#### 2.2.4. Color Measurements of Cookies

Color measurements were carried out using a colorimeter (Konica Minolta Sensing, Inc., CR-400, Osaka, Japan) and expressed as L* (darkness/lightness), a* (redness/greenness), and b* (yellowness/blueness) values. The colorimeter was calibrated to a white calibration plate (CR-A44 Konica Minolta Sensing Inc., Osaka, Japan) before use. Color measurement was determined in triplicate. The color intensity (ΔE) and ΔL (Equation (1)) were calculated according to the following equation [20,21].

(1) $$\Delta E=\sqrt{{\left(\Delta L\right)}^{2}+{\left(\Delta a\right)}^{2}+{\left(\Delta b\right)}^{2}}$$

where ΔL = L_sample_ − L_control_; Δa = a_sample_ − a_control_; Δb = b_sample_ − b_control._

#### 2.2.5. Texture Profile Analysis

Texture profile analysis (TPA) of cookie dough was performed using a TA-TX plus Texture Analyser (Stable Micro Systems, Surrey, UK), according to a developed method by Kumar et al. [22]. For TPA, pre-test speed, post-test speed, and test speed were each arranged at 2 mm/s, and the compression was 50% of the height of the cookie dough. An interval of 5 s between two compression cycles and a trigger force of 0.5 g were selected. A P/36 cylindrical probe was used for TPA.

Texture profile analysis (TPA) of cookie samples was performed using a TA-TX plus Texture Analyser (Stable Micro Systems, Surrey, UK) equipped with a 3-point bending rig, which was used for texture analysis, and the maximum force (Newton, N) required to break the cookie sample was determined 24 h after baking. For TPA, pre-test speed, test speed, and post-test speed were each arranged at 1 mm/s, 3 mm/s, and 10 mm/s. An interval of 5 s between two compression cycles and a trigger force of 50 g were selected [23].

#### 2.2.6. Determination of Phenolic Contents (Free, Bound, and Total) and Antioxidant Capacities

Free and bound phenolic compounds of the cookie samples were extracted according to Ozkan et al. [24], with slight modifications. Defatted cookie samples (0.5 g) were mixed with 5 mL of a methanol–water solution (80:20 *v*/*v*) to extract phenolic compounds. The extraction was performed for 1 h in a shaking water bath at 200 rpm and 25 °C. After extraction, the mixture was centrifuged at 2500× *g* for 10 min at 4 °C. This extraction procedure was repeated three times. The supernatants were carefully collected with a pipette, combined in a separate tube, and stored at 4 °C. The remaining solid residue (pellet) was placed in a drying oven at 30 ± 2 °C with the lid open and left overnight.

The remaining solid residue (pellet) was subjected to alkaline hydrolysis using 20 mL of 2 N NaOH and incubated in a shaker at 200 rpm for 4 h. Following hydrolysis, the pH of the mixture was adjusted to 1.8–2.2 using 6 M HCl. To remove residual free fatty acids, liquid–liquid extraction with hexane was performed two times. The hexane layer was carefully removed using a pipette after each extraction. Subsequently, bound phenolics were extracted using 10 mL of a diethyl ether–ethyl acetate mixture (1:1, *v*/*v*). The mixture was vortexed, shaken at 200 rpm for 15 min, and centrifuged at 2500× *g* for 8 min. The organic (diethyl ether–ethyl acetate) layer was collected with a pipette, and this extraction process was repeated five times. The solvents in the combined supernatants were then evaporated using a rotary evaporator. The free and bound fractions were dissolved in 5 mL of methanol and stored at −18 °C until analysis.

To determine the concentrations of free and bound phenolic compounds, a modified Folin–Ciocalteu method was used. The Folin–Ciocalteu method was used to determine concentrations of free and bound phenolic compounds [24]. The results are given as mg gallic acid equivalent (GAE) per 100 g on a dry basis (db). The total phenolic content (TPC) was calculated as the sum of free and bound phenolics. ABTS, DPPH, and FRAP antioxidant capacities were determined according to the methods described in Ozkan et al. [24]. These results were expressed as mg Trolox equivalent (TE) per 100 g db.

#### 2.2.7. In Vitro Glycemic Index Value

The starch hydrolysis rate during in vitro digestion at 90 min and the in vitro glycemic index (GI) value of the cookie samples were measured according to the method of Kahraman et al. [23] by using a Glucose Assay Kit (Megazyme Int., Wicklow, Ireland), with gastric and intestinal digestion phases conducted in a water bath incubated at 37 °C. The in vitro GI was determined by using the following equation, Equation (2) of Kahraman et al. [23].

(2) $$GI=39.71+\left(0.549\times HI\right)$$

#### 2.2.8. Statistical Analysis

Each experiment was conducted in a completely randomized design with at least three replicates per group. The obtained data were subjected to one-way analysis of variance (ANOVA) using the IBM SPSS Statistics^®^ (version 24; IBM Corp., Armonk, NY, USA) program, followed by the Tukey test at a 5% significance level (*p* ≤ 0.05). Furthermore, statistical differences of the glycemic index analysis data between sucrose- and xylitol-containing samples were determined with the *t*-test (*p* ≤ 0.05) using the same statistical software.

## 3. Results

### 3.1. Physical Properties of Cookies

The physical properties (width, thickness, and spread ratio) of colored wheat cookie samples formulated with sucrose and xylitol are presented in Table 1.

The width of cookies prepared with sucrose or xylitol changed between 6.64 and 7.03 cm and 6.60 and 6.71 cm, respectively. The results indicated that the best width results in two groups were determined in the sucrose-containing cookie samples, and xylitol slightly reduced the width of cookies compared to sucrose. The colored wheat flours notably influenced cookie geometry by decreasing width and increasing thickness compared to the control cookies, independent of the sweetener. This is an expected outcome since blends (50:50) of whole wheat flours of the colored wheats with cookie flour were used in cookie formulations. Utilization of whole wheat flours in cookie formulation is expected to decrease the width and increase the thickness of cookies with their higher dietary fiber content [24]. In the sucrose-containing group, the control cookie had the highest width (7.03 cm) and the lowest thickness (1.17 cm) values, while in the xylitol-containing group, the purple wheat cookie had the highest width (6.71 cm) and the lowest thickness (1.24 cm) values. The width of the purple wheat cookie was even higher than that of the respective control cookie.

In terms of thickness, sucrose-containing cookies revealed a thinner range (1.17 cm to 1.31 cm) compared to xylitol-containing cookies (1.24 cm to 1.35 cm). It was also previously reported that sugar alcohols such as xylitol decrease cookie spread due to their lower hygroscopicity and different melting behavior [25]. The blue wheat cookies, including xylitol and sucrose, had the same thickness. The lowest width result was observed in black wheat cookie (6.64 cm) in the sucrose-containing group, while in the xylitol-containing ones, the lowest width value was observed in red wheat cookies (6.60 cm). The highest thickness values were observed in the cookie samples produced using red wheat (1.35 cm) and black wheat (1.31 cm) for the xylitol- and sucrose-containing groups, respectively.

The spread ratios of the xylitol-containing cookies generally decreased compared to the respective sucrose-containing ones, except for the cookies produced from black wheat (5.07 vs. 5.12). For the sucrose-containing group, the control sample had the highest spread ratio, while in the xylitol-containing group, the purple wheat cookie sample had the highest spread ratio (5.41).

### 3.2. Color Development in Cookie Doughs and Cookies

Color is a key quality attribute in cookies, influenced by both ingredients, such as the color of wheat flour, and baking conditions. The colorimetric comparison of the cookie dough and baked cookies produced using colored wheat varieties and alternative sweetener (xylitol) reveals how baking transforms color parameters L* (lightness), a* (redness), and b* (yellowness). By using L*, a*, and b* values, ΔL and ΔE (color intensity) parameters were obtained. These parameters also consolidate the analysis of the visual impact of colored wheat and the baking process. The color parameters of the cookie doughs prepared with colored wheat are given in Table 2.

The following discussion will first examine the colorimetric differences of the dough samples, then the baked samples, including the influence of the sweeteners. There were significant differences in the color values of the cookies (*p* ≤ 0.05). Control doughs produced from white cookie flour displayed the highest L* values, indicating the lightest color (63.81 and 67.83 for sucrose- and xylitol-containing cookies, respectively), while the doughs including pigmented wheat flours appeared darker, as reflected by their noticeably reduced L* values. Among the sucrose-containing dough samples, black wheat dough exhibited the lowest L* (41.78), consistent with its darker appearance.

The a* values of red and purple wheat doughs with both sweeteners were markedly high, depicting more redness compared to the control samples. On the other hand, blue wheat dough had a slightly positive a* value, showing its bluish-gray color.

The addition of pigmented wheat flours reduced b* values, especially for blue and black wheat doughs, reflecting their less yellow and paler tones. The a* values of red and purple wheat doughs with both sweeteners were markedly high, depicting more redness compared to the control samples. On the other hand, blue wheat dough had a slightly positive a* value, showing its bluish-gray color. The addition of pigmented wheat flours reduced b* values, especially for blue and black wheat doughs, reflecting their less yellow and paler tones.

Determining ΔL and ΔE values further emphasized the differentiation between control and colored wheat doughs. The highest ΔE (overall color difference) values were obtained in black (28.28) and blue wheat (26.83) cookies, including xylitol, indicating obvious color deviation from the control. The pronounced color change in black and blue wheat doughs might be related to their higher anthocyanin content [8].

All of the xylitol-based doughs demonstrated higher lightness (L*) values compared to their counterparts prepared with sucrose, which may be due to the inherently white, crystalline nature of xylitol. Previous research also confirmed that polyol sweeteners such as xylitol increase the L* value, resulting in a brighter dough relative to sucrose formulations [17]. In Table 3, the color parameters of the cookie samples prepared with colored wheats are given.

The lightness (L*) and yellowness (b*) values of the cookies produced from colored wheats were generally lower than those of the respective control cookies, while their redness (a*) values tended to be higher, regardless of the type of sweetener used.

**Figure 1:**
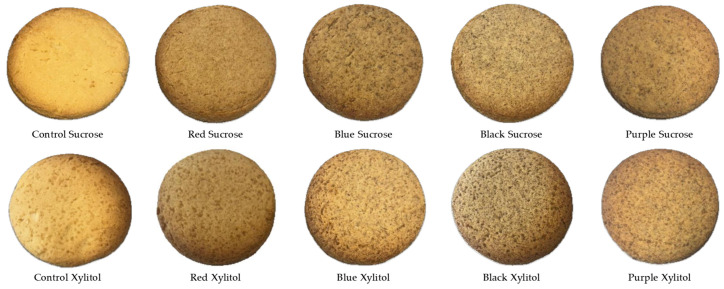
Images of cookie samples with added sucrose or xylitol prepared using colored whole wheat flours.

Baking significantly impacted the color parameters (L*, a*, b*) of the cookies, but the relative color order among different samples was generally preserved. Notably, L* values were elevated for all samples compared to dough, indicating a lightening effect or surface reflection. This phenomenon is linked to moisture loss and the transition from opaque, moist dough to a drier, more reflective surface [26,27]. Images of cookie samples are shown in Figure 1.

Control cookies maintained the brightest L* values (68.38 and 69.07 for sucrose and xylitol), whereas black (54.20, 52.31 for sucrose and xylitol) and blue wheats (56.98, 56.69 for sucrose and xylitol) remained the darkest.

After baking, both a* and b* values increased for most samples, reflecting the progression of Maillard and caramelization reactions that intensify color development [28]. This effect was more pronounced in sucrose-containing cookies than in xylitol-containing ones, conforming with the higher browning potential of sucrose [16]. Sucrose is not a reducing sugar; however, it breaks down to constituent monosaccharides (fructose and glucose) upon heating. These reducing sugars participate in Maillard reactions. Sucrose also goes through caramelization at baking temperatures and produces a brown color [28].

Among sucrose-containing cookies, red and purple wheat cookies demonstrated significantly higher a* values (6.58 and 7.33), reflecting intensified reddish-brown tones, whereas blue and black wheat cookies maintained lower a* and b* values due to their darker appearance, masking the color developed during browning reactions.

ΔE values between control and colored wheat cookies ranged from 6.44 to 19.78. The differences were smaller than in doughs, implying that the baking process partially decreased the visual differences of colored wheat cookies. This could be due to the general browning of all samples during baking, which reduced the contrast. However, black wheat cookies prepared with xylitol still exhibited the highest ΔE (19.78), indicating that their general appearance (probably due to anthocyanins) retained a strong influence even after baking [29].

### 3.3. Textural Characteristics of the Cookie Dough and Cookie Samples

The textural properties of cookie doughs and cookies prepared using xylitol or sucrose and blends of colored wheat flours (red, blue, purple, and black) with the cookie flour are given in Table 4.

The cookie doughs containing sucrose had higher hardness values compared to the ones containing xylitol. The black cookie dough containing sucrose (56.69 N) or xylitol (41.82 N) had the hardest values in their group. The lowest hardness values were obtained in the control samples for both sugar groups, 32.56 N for sucrose-containing controls and 24.02 N for xylitol-containing controls.

Sugar competes with flour for the water that is present, causing fewer gluten bonds to form and making the cookie less cohesive as a result. Sugar may either help make the cookie crispier through crystallization or soften the cookie by taking up water [30]. The solubility of sucrose and xylitol in water at 25 °C is 210 g and 200 g per 100 mL of water, respectively [31]. Xylitol, having greater solubility, results in a softer dough. The adhesiveness in the cookie dough is associated with the sugar that dissolves before baking. For this reason, the high solubility of sugar during dough formation increases its adhesiveness. When sugar dissolves, the total solution volume increases within the dough, affecting dough consistency and machinability. Dissolved sugar creates a syrup-like environment and inhibits the formation of the gluten network. Therefore, more adhesive dough is formed [32]. Lower cohesiveness values indicate that the dough is crumbly or brittle. The development of the gluten network is largely responsible for the structure and cohesiveness of bakery products. High extensibility/springiness is not a desirable property for a cookie dough [32].

According to Rodriguez-Garcia et al. [33], highly concentrated sugar solutions limit molecular mobility, which delays gluten network formation during mixing and increases the gelatinization temperature of starch during baking. The resulting syrup also modifies dough consistency and processing behavior while controlling gluten cross-linking and consequently influences dough spreading and structure development during baking.

Hardness is defined as the force required to cause the cookie to break completely, while brittleness is expressed as an indicator of the fragility and tendency of the cookie to crumble [34]. Table 4 also summarizes the textural properties of the baked cookies based on hardness and brittleness factors. Among the sucrose- and xylitol-containing cookies, the lowest hardness values (10.55 and 5.45 N, respectively) were observed in the control samples. The sucrose-containing control cookie had the lowest brittleness value (1.59 mm), while in the xylitol-containing group, the purple wheat cookie had the lowest brittleness value (1.41 mm). The control sample had the second-lowest brittleness value in the xylitol-containing group (1.65 mm).

With both types of sweeteners used, the highest brittleness values were observed in the sample produced using blue wheat, which were determined as 2.04 mm for sucrose and 2.10 mm for xylitol-containing cookies. The highest hardness values were found in the purple wheat cookie (14.68 N) in the sucrose-containing samples and the black wheat cookie (9.27 N) in the xylitol-containing samples. Among the xylitol-containing group, the red wheat cookie stands out among all cookies with the lowest hardness value (6.40 N), except the control cookie.

Koksel et al. [14] reported that total dietary fiber (TDF) contents of whole wheat flours of red, purple, and blue wheats were in the range of 17.2 to 19.3%, and TDF of their breads was in the range of 19.5 to 19.7%, indicating relatively high TDF contents of colored whole wheat flours. Since the blends (50:50) of whole wheat flours of the colored wheats with cookie flour were used in cookie formulations in the present study, their TDF contents were expected to be relatively high. Dhal et al. [35] stated that high fiber content of whole wheat flour can increase cookie hardness. They rationalized this with the following explanation: fiber can make cookies denser and crunchier by absorbing water, and they noted that too much fiber could result in dry, crumbly cookies. Nicetin et al. [34] also reported that the increase in cookie hardness was directly proportional to the increase in the addition level of high fiber celery root powder, and the hardness values were significantly higher for the samples containing higher levels of celery root powder compared to the control cookie. Therefore, higher hardness values of cookies produced from blends of whole wheat flours of the colored wheats compared to the control cookie produced from refined flour are in line with the related literature.

The results also indicated that sugar type has a significant effect on the hardness values of cookie samples. Rodriguez-Garcia et al. [33] reported that sugar crystals formed during baking are partially responsible for the hard and crisp texture of cookies. A previous study by Rutkowska et al. [16] reported that replacement of sucrose with xylitol altered the sensory properties, and cookies with added xylitol were about two-fold less hard than those with added sucrose in their fresh form. Hence, the lower hardness values of the xylitol-containing cookies compared to the sucrose-containing ones are in line with the related literature.

### 3.4. Phenolic Compounds and Antioxidant Capacities of Cookies

Phenolic contents (free, bound, and total) and antioxidant capacities (ABTS, FRAP, DPPH) of colored wheat cookies are given in Table 5. There were significant differences in phenolic contents of the cookies (*p* ≤ 0.05).

Phenolic compounds in foods occur either in free form or as covalently bound forms, including esters and ethers. These are referred to as insoluble-bound phenolics [36]. The free and bound phenolics of the cookies for the sucrose-containing group varied from 132.87 to 197.96 and 179.55 to 234.84 mg GAE/100 g db, respectively, while the free and bound phenolics of the cookies for the xylitol-containing group changed between 127.34 and 197.22 and 178.94 and 230.89 mg GAE/100 g db, respectively. As expected, in the present study, the amounts of bound phenolics in cookie samples were higher than the amounts of free phenolics. In cereal-based matrices, the majority of phenolic compounds occur in insoluble bound forms, which can be quantified through the analysis of hydrolysable fractions.

Bound phenolics may interact with starch and other macromolecules in the cookie matrix, influencing water retention, gelatinization behavior, and overall physicochemical properties, which in turn can affect dough structure and antioxidant capacity. Such starch–phenolic interactions, including hydrogen bonding and complex formation, have been shown to alter starch microstructure, competitiveness for free water, and thermal stability, thereby impacting functional and technological properties of starch-based foods [36].

Similar results were obtained by Dundar [37]; the bound fraction (hydrolysable) of the Bee pollen-enriched cookies was higher than the free fraction (extractable). In the present study, the total phenolic content (TPC) showed significantly higher values (highest—424.87 28 mg GAE/100 g db) in all colored wheat cookies than in the control cookies (306.28 and 312.42 mg GAE/100 g db). The TPC of purple wheat cookies prepared with both sucrose and xylitol was the highest within each sugar group, followed by blue wheat cookies. A similar result was observed by Koksel et al. [14], who reported that the breads prepared with purple whole wheat had the highest total phenolic content. Nignpense et al. [38] reported that purple wheat had greater phenolic content and antioxidant activity than blue wheat after cooking. In a study by Ozkan et al. [24], the TPCs of the red and black wheat breads were significantly lower compared to the blue wheat bread.

In the present study, three methods (DPPH, ABTS, and FRAP) were used to determine antioxidant capacity (Table 5). There were significant differences in antioxidant capacity values of the cookies produced from the colored wheat genotypes (*p* ≤ 0.05). Similar to the phenolic compound results, DPPH, ABTS, and FRAP values for bound fractions were higher than those of the free fractions. The free DPPH values of the cookies prepared with sucrose and xylitol ranged from 88.33 to 124.89 and 84.19 to 115.25 mg TE/100 g db, respectively, whereas the free FRAP values of the cookies prepared with sucrose and xylitol were between 5.01 and 38.20 and 4.15 and 37.95 mg TE/100 g db, respectively. Similar to the phenolic compound results, the antioxidant capacity values (DPPH, ABTS, and FRAP) of purple wheat cookies prepared with both sucrose and xylitol were the highest among other cookie samples. Purple wheat grains and their derived food products exhibit higher antioxidant capacity, largely attributed to their anthocyanin content and other phenolic compounds. In the present study, the DPPH values of cookies were higher than their ABTS values. A study by Heredia-Sandoval et al. [39] suggested that the antioxidant capacity was higher when using DPPH compared to the ABTS assay and that this could be due to differences in free radical scavenging mechanisms. The study attributed this difference to the fact that the DPPH scavenging assay only involves electron transfer, while the ABTS assay includes hydrogen atom transfer in addition to electron transfer. In line with the present study, Ozkan et al. [24] and XueFeng et al. [40] found that the antioxidant capacity of blue, black, and purple wheat grains was significantly higher compared to that of white and red wheat grains. According to Geyik et al. [13], the ABTS antioxidant capacity of bound extracts was 100.10 mg TE/100 g in red wheat, 207.49 mg TE/100 g in blue wheat, and 239.96 mg TE/100 g in purple wheat, whereas the free fractions showed lower values of 52.70, 87.77, and 76.87 mg TE/100 g, respectively. Koksel et al. [14] reported that the ABTS and DPPH values of the purple whole wheat bread were higher than those of the blue and red whole wheat bread.

Colored wheats (red, blue, purple, and black) contain higher amounts of anthocyanins and phenolic compounds compared to traditional white wheat, resulting in a richer functional profile [41]. Similar results were obtained in the present study; the control cookie (white cookie flour without bran) exhibited lower phenolic content and antioxidant capacity than the cookies prepared using 50:50 blends of colored whole wheat flours and white cookie flour.

### 3.5. The In Vitro Glycemic Index Values of Cookie Samples

Foods can be classified as having a low (GI < 55), medium (GI 55–70), or high (GI > 70) glycemic index according to their GI values [42]. The hydrolysis index and in vitro GI values of the cookie samples are given in Table 6. As expected, the highest hydrolysis index and in vitro GI (73.74 and 67.12, respectively) values were determined in the control cookie samples.

In contrast, the lowest hydrolysis indices and in vitro GI values were found in the cookie samples produced using blue wheat flour in both sugar groups, and these values were significantly lower compared to the cookies produced from other colored wheats within each sugar group (*p* ≤ 0.05). The GI values of blue wheat samples were determined as 66.68 and 60.94 for sucrose- and xylitol-containing cookies, respectively. These values belong to the medium glycemic index range.

In terms of GI values, although statistically significant differences were obtained among cookies containing xylitol, there were no significant differences among the GI values of sucrose-containing cookies produced using colored wheats, except for the one produced using the blue wheat. GI values of sucrose-containing colored wheat cookies were in the high GI range, except for the one from blue wheat, which was in the medium GI range. To evaluate the effect of different types of sugars on the glycemic index, the *t*-test was applied to cookies produced using the same-colored wheat flour. Statistical differences between the GI values of sucrose- and xylitol-containing samples were determined, and the results indicated the positive effect of xylitol on lowering the GI value.

As the xylitol-containing colored wheat cookie group was examined; black wheat had the highest GI (65.80), and the red and purple wheats’ GI values were determined as 64.38 and 62.96, respectively. These values were also in the medium GI range. In line with the present study, Koksel et al. [12] estimated the in vitro GI values of colored (red, purple, blue, black) whole wheat bread samples and found the lowest GI (63.64) in blue and the highest GI (72.03) in black wheat samples. Whole wheat products, with their high fiber content, slow down digestion, regulate post-meal blood sugar spikes, and increase feelings of fullness. Regular consumption of whole wheat foods is reported to be closely associated with a reduced risk of cardiovascular disease and type 2 diabetes [43,44].

Islam [45] reported that xylitol has a significantly lower glycemic index (13) compared with sucrose (65) and glucose (100). This study also revealed that xylitol, in addition to its blood glucose-lowering effect, significantly improved glucose tolerance levels in non-diabetic rats compared to sucrose. In a study conducted by Hassinger et al. [46], it was reported that the amount of insulin needed to control blood sugar in insulin-dependent diabetic patients was significantly lower for xylitol compared to starch or sucrose containing the same number of calories.

In a clinical study conducted by Su-Que et al. [42], the consumption of steamed bread produced using purple and white grain types was compared according to its effect on GI and postprandial plasma glucose in healthy and type 2 diabetic subjects. Bread made using purple wheat flour was found to lower the GI and attenuate the postprandial plasma glucose level. Phenolic compounds may slow starch digestion through several mechanisms: (i) inhibition of digestive enzymes via binding to their active sites; (ii) competition with glucose for intestinal transport, thereby reducing glucose absorption; and (iii) formation of crosslinked networks with starch during cooking, which physically limit amylolytic access and slow starch hydrolysis [47]. These effects may contribute to reduced glycemic responses in vivo. In line with at least some of these, the cookies produced using colored wheat flours had higher phenolic content and antioxidant capacity values compared to respective controls within each sugar group, and their GI values were also lower compared to the control cookies.

In the present study, total and bound phenolic contents as well as antioxidant capacities were highest in blue and purple wheat cookies. These elevated phenolic levels corresponded with medium GI values compared to other wheat varieties, suggesting potential mechanistic interactions between phenolic compounds and starch and the modulation of postprandial glucose response. Sun and Miao [48] have similarly reported that cereals with higher phenolic content exhibit slower starch digestion and reduced glycemic responses, supporting the mechanistic role of phenolics in glycemic modulation.

It should be noted that the glycemic index values presented in this study were calculated based on an in vitro starch hydrolysis assay and, consequently, reflect estimated GI values rather than true in vivo glycemic responses. Although this approach is widely accepted for comparative purposes, it does not fully reflect the complexity of human digestion and metabolic regulation. Consequently, further validation through controlled human studies is warranted to confirm the physiological relevance of the observed effects.

## 4. Conclusions

This study demonstrated that the use of colored wheat flours (red, blue, purple, and black) and the replacement of sucrose with xylitol significantly influenced the technological, physical, and nutritional properties of cookies. The physical characteristics of the cookies were affected by both flour type and sweetener. Cookies prepared with blends of colored whole wheat flours generally showed reduced width and increased thickness compared with the control cookies produced from refined flour. This behavior can largely be attributed to the higher dietary fiber content of whole wheat flours, which limits dough spread during baking. Similarly, the substitution of sucrose with xylitol generally decreased the spread ratio, which is consistent with its different hygroscopic and melting properties. Despite these differences, the physical characteristics of the cookies remained within acceptable ranges for typical cookie products.

Intrinsic pigmentation of colored wheat flours significantly affected the appearance of both doughs and baked cookies. Doughs containing colored wheats exhibited lower lightness values than the control, and these color differences were largely preserved after baking despite browning reactions. Cookies produced with black and blue wheats appeared darker, while red and purple wheats showed higher redness values. Additionally, xylitol-containing doughs displayed higher lightness than sucrose-based doughs due to the lower browning potential of xylitol during baking.

Both flour composition and sweetener type influenced dough and cookie texture. Doughs prepared with sucrose were harder than those containing xylitol, while the use of colored whole wheat flours increased dough firmness compared with the control. Similarly, cookies produced with colored wheats exhibited higher hardness and brittleness due to their higher dietary fiber content. However, replacing sucrose with xylitol resulted in cookies with lower hardness values.

The incorporation of colored wheat flours significantly enhanced the functional properties of cookies. All colored wheat cookies showed higher total phenolic content and antioxidant capacity compared with the control samples. The purple wheat cookies exhibited the highest phenolic content and antioxidant activity. In addition, the majority of phenolic compounds were present in the bound fraction, which is typical for cereal-based matrices. The in vitro glycemic index results further supported the nutritional advantages of the formulations. Control cookies exhibited the highest estimated GI values, whereas cookies produced with colored wheat flours showed lower GI values. Blue wheat cookies had the lowest GI values among the samples. The use of xylitol also contributed to a reduction in GI values.

Overall, the findings indicate that colored wheat flours combined with alternative sweeteners such as xylitol can be successfully utilized to produce cookies with improved antioxidant potential and lower glycemic response while maintaining acceptable technological quality. Future studies should focus on sensory evaluation, consumer acceptance, and long-term storage stability of such cookies, as well as the exploration of additional bioactive compounds and their bioavailability in colored wheat-based products.

## Figures and Tables

**Table 1:** Physical characteristics of cookies.

Sample	Width (cm)	Thickness (cm)	Spread Ratio
C/S	7.03 ± 0.03 ^a^	1.17 ± 0.03 ^e^	6.00 ± 0.03 ^a^
Red/S	6.70 ± 0.00 ^b^	1.28 ± 0.02 ^c^	5.27 ± 0.01 ^c^
Blue/S	6.73 ± 0.03 ^b^	1.29 ± 0.03 ^b^	5.21 ± 0.02 ^d^
Black/S	6.64 ± 0.03 ^c^	1.31 ± 0.03 ^a^	5.07 ± 0.01 ^e^
Purple/S	7.01 ± 0.02 ^a^	1.21 ± 0.03 ^d^	5.82 ± 0.03 ^b^
C/X	6.68 ± 0.03 ^ab^	1.26 ± 0.02 ^d^	5.30 ± 0.03 ^b^
Red/X	6.60 ± 0.04 ^b^	1.35 ± 0.03 ^a^	4.91 ± 0.03 ^e^
Blue/X	6.68 ± 0.05 ^ab^	1.29 ± 0.02 ^c^	5.19 ± 0.03 ^c^
Black/X	6.63 ± 0.03 ^b^	1.30 ± 0.02 ^b^	5.12 ± 0.02 ^d^
Purple/X	6.71 ± 0.02 ^a^	1.24 ± 0.03 ^e^	5.41 ± 0.02 ^a^

Data are expressed as mean ± standard deviation, and the statistical analysis was performed separately within each group containing sucrose (S) and xylitol (X). The values in each column with different superscript letters (a–e) are significantly different (*p* ≤ 0.05). C = control.

**Table 2:** Color analysis of cookie dough samples.

Sample Code	L*	a*	b*	ΔE
C/S	63.81 ± 0.37 ^a^	−0.39 ± 0.02 ^e^	29.14 ± 0.72 ^a^	-
Red/S	48.93 ± 0.66 ^c^	5.68 ± 0.23 ^a^	24.33 ± 0.31 ^b^	16.76
Blue/S	45.56 ± 0.81 ^d^	0.22 ± 0.05 ^d^	15.58 ± 0.05 ^d^	22.77
Black/S	41.78 ± 0.81 ^e^	2.83 ± 0.11 ^c^	13.96 ± 0.38 ^e^	26.77
Purple/S	51.57 ± 0.72 ^b^	4.40 ± 0.29 ^b^	19.02 ± 0.86 ^c^	16.58
C/X	67.83 ± 0.87 ^a^	−0.75 ± 0.12 ^e^	30.46 ± 0.36 ^a^	-
Red/X	52.16 ± 0.03 ^c^	5.92 ± 0.29 ^a^	25.68 ± 0.56 ^b^	17.34
Blue/X	45.69 ± 0.27 ^d^	0.05 ± 0.23 ^d^	16.16 ± 0.22 ^d^	26.83
Black/X	44.17 ± 0.23 ^e^	2.87 ± 0.08 ^c^	14.60 ± 0.42 ^e^	28.28
Purple/X	53.36 ± 0.08 ^b^	4.71 ± 0.25 ^b^	18.62 ± 0.23 ^c^	19.97

Data are expressed as mean ± standard deviation, and the statistical analysis was performed separately within each group containing sucrose (S) and xylitol (X). The values in each column with different superscript letters (a–e) are significantly different (*p* ≤ 0.05). C = control; L* = lightness/darkness; a* = redness/greenness; b* = yellowness/blueness; ΔE = overall color difference.

**Table 3:** Color analysis of cookie samples.

Sample Code	L*	a*	b*	ΔE
C/S	68.38 ± 0.09 ^a^	3.77 ± 0.12 ^d^	33.71 ± 0.45 ^a^	-
Red/S	63.18 ± 0.38 ^b^	6.58 ± 0.14 ^b^	31.20 ± 0.37 ^b^	6.44
Blue/S	56.98 ± 0.09 ^c^	2.86 ± 0.09 ^e^	23.79 ± 0.34 ^d^	14.93
Black/S	54.20 ± 0.11 ^e^	4.51 ± 0.14 ^c^	22.17 ± 0.71 ^e^	18.20
Purple/S	56.00 ± 0.41 ^d^	7.33 ± 0.10 ^a^	28.76 ± 0.44 ^c^	14.20
C/X	69.07 ± 0.45 ^a^	4.48 ± 0.26 ^c^	35.10 ± 0.58 ^a^	-
Red/X	60.74 ± 0.35 ^b^	6.51 ± 0.04 ^a^	30.20 ± 0.88 ^b^	9.89
Blue/X	56.69 ± 0.08 ^d^	3.27 ± 0.43 ^d^	23.83 ± 0.19 ^e^	16.12
Black/X	52.31 ± 0.41 ^e^	5.67 ± 0.05 ^b^	25.62 ± 0.14 ^d^	19.78
Purple/X	57.58 ± 0.03 ^c^	6.21 ± 0.02 ^ab^	28.00 ± 0.65 ^c^	13.73

Data are expressed as mean ± standard deviation, and the statistical analysis was performed separately within each group containing sucrose (S) and xylitol (X). The values in each column with different superscript letters (a–e) are significantly different (*p* ≤ 0.05). C = control; L* = lightness/darkness; a* = redness/greenness; b* = yellowness/blueness; ΔE = overall color difference.

**Table 4:** Textural properties of cookie dough and cookie samples.

Sample Code	Dough				Cookie	
	Hardness (N)	Adhesiveness	Springiness	Cohesiveness	Hardness (N)	Brittleness (mm)
C/S	32.56 ± 0.13 ^e^	−1.93 ± 0.03 ^d^	0.65 ± 0.05 ^b^	0.19 ± 0.02 ^b^	10.55 ± 0.36 ^e^	1.59 ± 0.00 ^e^
Red/S	53.33 ± 0.65 ^b^	−1.34 ± 0.07 ^b^	0.41 ± 0.01 ^c^	0.20 ± 0.01 ^b^	11.94 ± 0.01 ^d^	1.86 ± 0.00 ^c^
Blue/S	45.43 ± 0.76 ^c^	−0.92 ± 0.01 ^a^	0.34 ± 0.01 ^c^	0.19 ± 0.01 ^b^	13.58 ± 0.15 ^b^	2.04 ± 0.00 ^a^
Black/S	56.69 ± 0.62 ^a^	−1.57 ± 0.02 ^c^	0.36 ± 0.00 ^c^	0.23 ± 0.01 ^ab^	12.67 ± 0.01 ^c^	2.00 ± 0.01 ^b^
Purple/S	35.53 ± 0.17 ^d^	−2.75 ± 0.05 ^e^	0.89 ± 0.01 ^a^	0.26 ± 0.01 ^a^	14.68 ± 0.06 ^a^	1.67 ± 0.01 ^d^
C/X	24.02 ± 0.50 ^e^	−2.78 ± 0.12 ^e^	0.41 ± 0.04 ^c^	0.22 ± 0.00 ^a^	5.45 ± 0.07 ^d^	1.65 ± 0.02 ^d^
Red/X	31.35 ± 0.59 ^c^	−1.28 ± 0.08 ^a^	0.40 ± 0.05 ^c^	0.19 ± 0.01 ^a^	6.40 ± 0.13 ^c^	1.91 ± 0.01 ^b^
Blue/X	34.79 ± 0.56 ^b^	−1.59 ± 0.00 ^b^	0.64 ± 0.01 ^b^	0.21 ± 0.01 ^a^	7.46 ± 0.00 ^b^	2.10 ± 0.01 ^a^
Black/X	41.82 ± 0.74 ^a^	−1.85 ± 0.00 ^c^	0.45 ± 0.01 ^c^	0.23 ± 0.01 ^a^	9.27 ± 0.20 ^a^	1.73 ± 0.00 ^c^
Purple/X	26.57 ± 0.46 ^d^	−2.27 ± 0.05 ^d^	0.82 ± 0.00 ^a^	0.23 ± 0.02 ^a^	7.73 ± 0.00 ^b^	1.41 ± 0.03 ^e^

Data are expressed as mean ± standard deviation, and the statistical analysis was performed separately within each group containing sucrose (S) and xylitol (X). The values in each column with different superscript letters (a–e) are significantly different (*p* ≤ 0.05). C = control.

**Table 5:** Phenolic content and antioxidant capacities (DPPH, FRAP, and ABTS methods) of the cookie samples.

	Sample Code	Phenolic Content	DPPH	FRAP	ABTS
Free	C/S	132.87 ± 0.51 ^e^	88.33 ± 0.50 ^e^	5.01 ± 0.48 ^d^	15.28 ± 0.45 ^e^
	Red/S	160.64 ± 0.88 ^d^	104.86 ± 0.06 ^d^	24.74 ± 0.64 ^c^	39.28 ± 0.45 ^d^
	Blue/S	188.57 ± 0.34 ^b^	112.03 ± 0.50 ^b^	29.60 ± 0.00 ^b^	84.16 ± 0.06 ^b^
	Black/S	182.40 ± 0.86 ^c^	106.83 ± 0.12 ^c^	31.06 ± 0.00 ^b^	66.44 ± 0.45 ^c^
	Purple/S	197.96 ± 1.32 ^a^	124.89 ± 0.61 ^a^	38.20 ± 0.47 ^a^	85.63 ± 0.06 ^a^
Bound	C/S	179.55 ± 0.67 ^e^	91.83 ± 0.50 ^e^	13.70 ± 0.01 ^d^	19.39 ± 0.78 ^e^
	Red/S	207.35 ± 0.76 ^d^	109.59 ± 0.19 ^c^	15.26 ± 0.01 ^d^	44.02 ± 0.45 ^d^
	Blue/S	234.81 ± 0.85 ^a^	118.42 ± 0.50 ^b^	34.83 ± 0.64 ^b^	89.03 ± 0.17 ^b^
	Black/S	230.01 ± 0.53 ^b^	106.30 ± 0.12 ^d^	23.95 ± 0.48 ^c^	69.12 ± 0.67 ^c^
	Purple/S	225.98 ± 0.10 ^c^	166.23 ± 0.61 ^a^	86.29 ± 0.47 ^a^	91.03 ± 0.27 ^a^
Total	C/S	312.42 ± 0.17 ^e^	180.16 ± 0.99 ^d^	18.70 ± 0.47 ^e^	34.67 ± 0.00 ^e^
	Red/S	367.41 ± 0.82 ^d^	214.53 ± 0.00 ^c^	40.01 ± 0.64 ^d^	83.30 ± 0.00 ^d^
	Blue/S	422.75 ± 0.30 ^b^	230.45 ± 0.00 ^b^	64.43 ± 0.64 ^b^	173.19 ± 0.11 ^b^
	Black/S	412.41 ± 0.15 ^c^	212.96 ± 0.00 ^c^	55.01 ± 0.48 ^c^	135.95 ± 0.56 ^c^
	Purple/S	424.69 ± 0.15 ^a^	291.12 ± 1.21 ^a^	124.49 ± 0.00 ^a^	176.66 ± 0.33 ^a^
Free	C/X	127.34 ± 0.77 ^e^	84.19 ± 0.25 ^e^	4.15 ± 0.16 ^e^	12.26 ± 0.45 ^e^
	Red/X	161.00 ± 1.23 ^d^	100.75 ± 0.78 ^d^	23.57 ± 0.03 ^d^	38.98 ± 0.70 ^d^
	Blue/X	184.91 ± 0.07 ^b^	109.37 ± 0.25 ^b^	25.05 ± 0.04 ^c^	74.22 ± 0.45 ^b^
	Black/X	181.30 ± 0.09 ^c^	104.71 ± 0.51 ^c^	25.99 ± 0.02 ^b^	61.83 ± 0.91 ^c^
	Purple/X	197.22 ± 1.06 ^a^	115.25 ± 0.52 ^a^	37.95 ± 0.33 ^a^	79.65 ± 0.70 ^a^
Bound	C/X	178.94 ± 0.34 ^e^	87.02 ± 0.25 ^e^	12.05 ± 0.04 ^e^	16.73 ± 0.45 ^e^
	Red/X	212.62 ± 0.80 ^d^	102.40 ± 1.04 ^d^	14.09 ± 0.01 ^d^	43.61 ± 0.70 ^d^
	Blue/X	230.89 ± 0.43 ^a^	110.35 ± 0.63 ^b^	30.50 ± 0.32 ^b^	75.87 ± 0.08 ^b^
	Black/X	224.11 ± 0.86 ^c^	105.60 ± 0.25 ^c^	23.46 ± 0.65 ^c^	63.52 ± 0.57 ^c^
	Purple/X	227.05 ± 0.66 ^b^	116.72 ± 0.52 ^a^	89.08 ± 0.83 ^a^	77.74 ± 0.12 ^a^
Total	C/X	306.28 ± 0.43 ^e^	171.21 ± 0.50 ^e^	16.20 ± 0.20 ^e^	29.00 ± 0.00 ^e^
	Red/X	373.62 ± 0.44 ^d^	203.14 ± 0.26 ^d^	37.66 ± 0.05 ^d^	82.09 ± 0.70 ^d^
	Blue/X	415.80 ± 0.36 ^b^	219.71 ± 0.38 ^b^	55.55 ± 0.28 ^b^	150.09 ± 0.53 ^b^
	Black/X	405.41 ± 0.77 ^c^	210.31 ± 0.76 ^c^	49.46 ± 0.67 ^c^	125.35 ± 0.34 ^c^
	Purple/X	424.87 ± 0.86 ^a^	231.98 ± 1.04 ^a^	127.02 ± 0.53 ^a^	157.39 ± 0.82 ^a^

Data (free, bound, and total phenolics/antioxidants) are expressed as mean ± standard deviation, and the statistical analysis was performed separately within each group containing sucrose (S) and xylitol (X). The values in each column with different superscript letters (a–e) are significantly different (*p* ≤ 0.05). Phenolic contents are expressed as mg GAE/100 g dry weight (db). The sum of free and bound antioxidant capacities is expressed as mg TE/100 g db. C = control.

**Table 6:** Hydrolysis index and in vitro glycemic index values of cookie samples.

Flour Type	Hydrolysis Index (S)	Hydrolysis Index (X)	In Vitro Glycemic Index (S)	In Vitro Glycemic Index (X)
Control	62.56 ± 0.82 ^a^	49.92 ± 0.06 ^a^	73.74 ± 0.90 ^a,A^	67.12 ± 0.03 ^a,B^
Red	56.98 ± 0.02 ^b^	45.14 ± 0.10 ^c^	70.95 ± 0.03 ^b,A^	64.38 ± 0.21 ^c,B^
Blue	49.49 ± 0.53 ^c^	38.67 ± 1.06 ^e^	66.68 ± 0.58 ^c,A^	60.94 ± 0.58 ^e,B^
Black	56.65 ± 0.01 ^b^	47.77 ± 0.11 ^b^	70.84 ± 0.04 ^b,A^	65.80 ± 0.24 ^b,B^
Purple	57.57 ± 0.01 ^b^	42.35 ± 0.06 ^d^	71.80 ± 0.04 ^b,A^	62.96 ± 0.03 ^d,B^

Values are expressed as mean ± standard deviation. Statistical differences between sucrose- and xylitol-containing samples were determined using an independent samples *t*-test (*p* ≤ 0.05). Different lowercase superscript letters (a–e) within the same column indicate significant differences among flour types. Different capital superscript letters (A–B) within the same row indicate significant differences between the cookies containing (S) sucrose and xylitol (X). C = control.

## Data Availability

The original contributions presented in this study are included in the article. Further inquiries can be directed to the corresponding authors.

## Abbreviations

The following abbreviations are used in this manuscript:

ABTS	2,2′-Azinobis-3-ethylbenzothiazoline-6-sulfonic acid
ANOVA	Analysis of variance
DPPH	2,2-Diphenyl-1-picrylhydrazyl
db	Dry basis
FRAP	Ferric ion reducing antioxidant potential
GI	Glycemic index
GAE	Gallic acid equivalent
HI	Hydrolysis index
L	Lightness
Inc	Incorporation
TDF	Total dietary fiber
TE	Trolox equivalent
TPC	Total phenolic content
